# RxTrends: An R-based Shiny Application for Visualising Open Data on Prescribed Medications in Ireland

**DOI:** 10.12688/hrbopenres.14080.2

**Published:** 2025-05-27

**Authors:** Ahmed Hassan Ali, Michelle Flood, Ciara Kirke, Molly Mattsson, Mary E. Walsh, Emma Wallace, Derek Corrigan, Tom Fahey, Fiona Boland, Frank Moriarty

**Affiliations:** 1 School of Pharmacy and Biomolecular Sciences, Royal College of Surgeons in Ireland (RCSI) University of Medicine and Health Sciences, Dublin, Leinster, Ireland; 2 National Medication Safety Programme, HSE National Quality and Patient Safety Directorate, Dublin, Dublin, Ireland; 3 Department of General Practice, University College Cork, Cork, County Cork, Ireland; 4 Health Technology Assessment Directorate, Health Information and Quality Authority, Dublin, Leinster, Ireland; 5 Department of General Practice, RCSI University of Medicine and Health Sciences, Dublin, Ireland; 6 Data Science Centre, RCSI University of Medicine and Health Sciences, Dublin, Ireland

**Keywords:** General Medical Services (GMS), Prescribing Pattern, Data Visualisation, Shiny application

## Abstract

**Background:**

The Health Service Executive (HSE) in Ireland releases monthly reports on prescription dispensing claims and payments relating to community drug schemes. This paper describes the implementation of an R-based Shiny application that facilitates interactive visualisation and analysis of trends in medication prescribing and improves the data’s practical value, and presents use cases focused on drug utilisation and medication policy questions.

**Methods:**

Using Primary Care Reimbursement Service (PCRS) data provided by the HSE relating to the means-tested General Medical Services (GMS) scheme covering approximately one-third of the population, an R-based Shiny application was developed. This application uses monthly prescribing and cost data from 2016 up to the most recent data available (currently October 2024) relating to the 100 most commonly prescribed medications (by frequency and cost) and all therapeutic groups. The application leverages a range of R packages to enable users to select medications, therapeutic groups, and physiological systems to explore and compare prescribing and cost trends over time.

**Results:**

The RxTrends Shiny application effectively integrates PCRS data, providing multiple functionalities that allow for visualisation of dispensing trends of multiple medications, therapeutic groups and physiological systems. Graphs are available across multiple prescribing frequency and cost metrics and can be restricted to a selected time period. The ‘compare’ function visualises the proportion of prescribing/cost a selected medication or therapeutic group accounts for within a therapeutic group or physiological system. Use cases relating to Ireland’s Preferred Drug Initiative, availability of generic products and reference pricing, and seasonality of drug utilisation are presented.

**Conclusion:**

The application provides an interactive interface for stakeholders to visualise and monitor prescribing patterns using data from monthly PCRS reports. The application increases access to and usability of PCRS data for various audiences for whom it may be of interest, including researchers, healthcare professionals, policymakers and the general public.

## Introduction

The Primary Care Reimbursement Service (PCRS) is a division of the Health Service Executive (HSE, the provider of public health and social care services in Ireland). The PCRS processes payments to primary care health professionals, who offer specified health services to the general public ^1^. The PCRS facilitates payments for community services through a wide range of administrative schemes and payment arrangements. In the area of medications, this includes the General Medical Services (GMS) scheme (which accounts for the highest volume of dispensing claims), Drugs Payment Scheme (DPS) and Long-Term Illness (LTI) scheme ^1, 2^.

GMS scheme eligibility is assessed based on income, expenses, age, marital status, and dependents, and provides benefits in terms of health cover to those who meet the criteria ^1, 3^. Eligible persons under the GMS scheme are entitled to receive free or reduced cost medical and surgical services and most prescribed medications and appliances from pharmacists ^1^. In 2016, 35.5% of the Irish population was covered by the GMS scheme, which decreased to 30.4% by 2022 ^4, 5^. The GMS scheme over-represents females, older persons and individuals with a reduced socioeconomic status. For instance, over 70% of the population age 70 years or older were GMS scheme-eligible in 2022 ^4^.

In 2022, the PCRS processed 19.4 million GMS prescription forms for payment, covering over 65.25 million prescription items which accounted for 71% of all items paid for by the PCRS ^2^. These dispensed items accounted for over 60% (€1,015,607,700) of the cost of all payments to pharmacists (mostly reimbursed ingredient costs, as well as dispensing fees and VAT where applicable) ^2^. This corresponds to an average expense of €15.47 for each dispensed item (excluding items dispensed for use in GP practices, such as dressings), with an average ingredient cost of €10.18 ^2^. Moreover, 87% of GMS scheme-eligible persons were dispensed prescription items, with an average annual expenditure of €740.91 per individual ^2^. In 2022, medications for the nervous system, alimentary tract and metabolism, and cardiovascular system had the highest prescribing frequency and cost expenditure ^2^.

Several countries regularly release open ( *i.e.* publicly available) data on prescribing, for example, the National Health Service (NHS) Scotland Open Data ^6^, NHS English Prescribing Dataset ^7^ and Netherlands' Medicines and Resources Information Project ^8^. These datasets represent potentially valuable resources for researchers, healthcare providers, and policymakers. However, identifying meaningful patterns in raw data can be challenging without effective visualisation tools. OpenPrescribing is an example of how this challenge can be addressed by both aggregating prescribing data and providing interactive, easily interpretable visualisation of the data ^9^.

OpenPrescribing was first launched in 2016 to facilitate analysis of NHS England primary care prescribing data ^10^. Through the development of the OpenPrescribing.net dashboards, users can easily navigate complex datasets, e.g. to explore specific medication trends both at the national level and across local Clinical Commissioning Groups (CCGs) ^9, 10^. OpenPrescribing supported researchers in assessing variation in clinical practice in the NHS, including prescribing trends and associated geographical variation ^11^. Additionally, NHS Digital datasets aggregated by OpenPrescribing have also been used in research evaluating behaviour changes in clinical practice, such as prescribers’ response to new guidelines and policies ^12^.

In Ireland, the PCRS also releases open data through monthly reports on prescriptions, claims, and payments, including structured tables listing the top 100 prescribed medications and all therapeutic groups ordered by prescribing frequency and cost. However, the utility of this data is limited by the lack of any platform or application that enables interactive visualisation and analysis. This paper describes an R-based Shiny application implementation to visualise trends in prescribing data from the GMS scheme reported by the PCRS in Ireland, offering dynamic and interactive exploration. It also provides several use cases which illustrate how this application can be used in practice. The rationale for developing this application was limitations identified in the utility of the raw PCRS data and lack of identified visualisation tools for these datasets, and was informed by the needs of knowledge users and reviewing functionality of similar tools such as OpenPrescribing.net.

## Method

### Data source and preparation

The HSE PCRS pharmacy claims data for Ireland was accessed via the PCRS’s Reporting and Open Data platform (available at https://www.sspcrs.ie/portal/annual-reporting/). We used their reports of monthly figures for the top 100 prescribed medications, ordered by their prescribing frequency or ingredient cost, as well as equivalent figures for total prescribing by therapeutic group, for the GMS scheme. We also used their reports on monthly numbers of eligible people for the GMS scheme. The current RxTrends application uses monthly prescribing data from January 2016 to October 2024 for the subset of the population who were eligible for the GMS scheme. GMS scheme data captures dispensing information for all reimbursed items for all eligible persons. We used this drug scheme for the initial development of this application, as for the Drugs Payment Scheme, data for any month only includes dispensings where household prescription medication expenditure exceeds a certain threshold. Additionally, Long Term Illness scheme data includes only medications that are related to specified health conditions, and only overall scheme eligibility is reported rather than by individual condition. Based on feedback following application launch, we also incorporated data from other drug schemes, however this paper focuses only on GMS scheme data where eligibility figures are reliable.

Downloading of data from the PCRS open data platform and initial processing were performed using JupyterLab, a web-based interactive computing environment for Python ( https://jupyter.org). All code and packages utilised are available in the Data Availability section.

The monthly data are structured in a tabular format, detailing the medication or therapeutic group name, total monthly frequency of their prescriptions, their percentage share of total GMS prescriptions for the selected month, the overall ingredient cost of these prescriptions in euros, and their percentage share of total GMS prescription cost. Data on monthly GMS scheme eligibility for 2017–2024 were also accessed through the PCRS’s Reporting and Open Data platform. Eligibility figures for 2016 were directly requested via email correspondence with the PCRS’s Reporting and Open Data team. The eligibility data detail the number of eligible persons in each Community Health Organisation and Local Health Office area, broken-down by gender and age groups. This application utilises the total eligible number in all areas for each month.

The prescriptions data categorised under therapeutic groups and physiological systems are coded according to the World Health Organization (WHO) Anatomical Therapeutic Chemical (ATC) Classification System ^13^. Additionally, ATC codes for medications were added to enhance the utility of the individual medications data. Therefore, this coding facilitates comparisons of related ATC codes at different levels. Certain medication groups under the "Various" physiological system (V) were reported as pharmacological subgroups ( *i.e.* 3rd level ATC) within both the top 100 medications data and the therapeutic groups data ( *i.e.* 2nd level ATC). These groups, such as urinary requisites, diagnostic agents, and needles, were excluded from the list of individual medications, and were only included in the application as part of the therapeutic group data. This was the more appropriate categorisation of these groups (as they were not individual medications), and the therapeutic group figures appear to provide a more comprehensive representation of the dispensing of these items.

Prescribing rates and cost rates were derived by divided values of prescribing frequency or cost of each medication, therapeutic group or physiological system by the total number of GMS scheme-eligible persons for the same month, and presented in rates per 1,000 eligible persons. The ingredient costs and related rate figures were also adjusted for inflation using the monthly Consumer Price Index (CPI), normalised to the most recent month in the data ( *i.e.* October 2024). The monthly CPI figures, which track the average price change of consumer goods and services purchased by private households, were sourced from the Central Statistics Office (CSO) of Ireland ^14^. In May 2021, A cyberattack on the health service of the Republic of Ireland resulted in transmission issues for pharmacy claims for that month ^15^. As a result, there was a notable fall in the apparent dispensing for that month and an increase for June 2021, as most data for dispensing in May were transmitted in June ^15^. The weighted mean of the metrics value ( *e.g.* prescribing, cost) for the two months was calculated for each month. If a record was only available for one month ( *i.e*. a medication was not part of the top 100 for one of the months), its value was utilised for that specific month and was not modified and applied to the other.

### Shiny application development

***Implementation***

An interactive web-based Shiny application was developed to enable dynamic visualisation and provide descriptive analysis of prescribing patterns using data sourced from the HSE-PCRS. The application leverages a variety of R packages, including Shiny for the web framework, ggplot2 and plotly for graphical representations, and dplyr for data manipulation. The application’s architecture is divided into two main components: the user interface (UI) and server-side logic. The UI provides various interactive elements such as dropdown menus with search function for selecting medications, therapeutic groups, and physiological systems, and a choice of slider or dropdown menu for selecting date ranges. The server-side logic processes the PCRS data which is initially fed from an Excel file using the readxl package.

Upon application launch, the UI dynamically populates selection choices based on the data. Users can select specific criteria to filter the data, which are then visualised through a series of tabbed panels displaying prescribing frequencies, costs, and rates (see Table 1) using plotly-enhanced ggplot2 charts. Each plot visualises trends over time, and users can interact with the data points for more detailed information. Additionally, the application includes custom JavaScript via shinyjs to enhance the UI elements like date range sliders, improving the user experience by formatting dates and updating labels dynamically.

**Table 1.** Description of the output tab panels in the RxTrends Shiny application.

Tab Panel	Description
Prescribing Frequency	It visualises the number of times a medication was dispensed per month and corresponds to the variable “Prescribing Frequency” in the source data.
Cost	It visualises the total ingredient cost of dispensings of a medication in euro per month and corresponds to the variable “Ingredient Cost” in the source data.
Cost per Prescribing	It visualises the mean ingredient cost per dispensing of a medication per month and is derived by dividing the “Ingredient Cost” variable by the “Prescribing Frequency” variable in the source data.
Prescribing Rate and Cost Rate	It visualises the number of dispensings/ingredient cost per month of a medication per 1,000 GMS eligible persons. They are derived by dividing the “Prescribing Frequency” or “Ingredient Cost” variables by the number of GMS eligible persons and multiplying by 1,000.

The RxTrends application's capabilities were extended by allowing users to select an unlimited number of entities across multiple categories, including medications, therapeutic groups, and physiological systems. The "Compare Selected" button in the application enables users to compare frequencies, costs, or rates over time when two distinct entities are selected. This feature allows the application's server-side logic to compute for the selected metric ( *e.g.* prescribing frequency, cost of the medication/therapeutic group) the proportion of higher-level entity in the WHO ATC classification hierarchy ( *i.e.* therapeutic group or physiological system) made up of the lower-level entity ( *i.e.* medication or therapeutic group). These comparisons are dynamically visualised in the interactive plots powered by ggplot2 and plotly.

The application also features a download button allowing users to download data containing the selected inputs currently displayed for further analysis or use. Downloadable data is provided in Comma-separated values (CSV) format, including columns for the metrics listed in Table 1 and one row per month per selected medication, therapeutic group, and/or physiological system. A contextual help and documentation feature has also been embedded within the application, which describes output data format in detail. 

During the development of RxTrends, the application underwent two refinement cycles, with team members (from clinical, epidemiology, and data science backgrounds) evaluating a prototype application and providing feedback on features, functionality, and presentation.

***Operation***

This RxTrends Shiny application was developed using R programming, version 4.3.3 ( www.r-project.org) for Microsoft Windows 10 x64, with the following attached base packages: stats, graphics, grDevices utils, datasets, methods, and base. Other attached packages were dplyr (1.1.4), lubridate (1.9.3), DT (0.33), plotly (4.10.4), ggplot2 (3.5.1), openxlsx (4.2.5.2), readxl (1.4.3), readr (2.1.5), shinyjs (2.1.0), htmltools (0.5.8.1), and shiny (1.8.1.1).

This Shiny application is available online on shinyapps.io ( https://frankmoriarty.shinyapps.io/RxTrends-RShiny/) and on the webpage for the project this work relates to ( https://cdrx-project.eu/?page_id=90) to enable users to run it embedded within the website. Additionally, users can run this application locally in RStudio either by downloading the code underlying application and associated data (see Data Availability section), or by running it directly from GitHub using the following R command: shiny::runGitHub(repo = 'RxTrends-RShiny', username = 'moriarty-pharmacoepi', ref = "master")

## Use cases

The RxTrends application can be used by those interested in exploring drug utilisation patterns, and potentially to facilitate drug utilisation research on questions across a range of domains. We present use cases to illustrate the potential for the application to explore questions relating to utilisation and costs.

### Prescribing frequency of preferred drugs in Ireland

The Preferred Drugs Initiative in Ireland aims to identify a certain drug within a therapeutic drug class as the preferred choice ^16^. This initiative offers prescribers clear guidelines on how to select, prescribe, and monitor the preferred drug for specific medical conditions ^16^. The selection of the drug is based on a comprehensive assessment of several aspects including clinical effectiveness, dosage, drug interactions, adverse effects, cost, national prescription patterns, and clinical recommendations ^16^. The HSE Medicines Management Programme has evaluated ten therapeutic classes and chosen a preferred drug within each, for example pantoprazole being the preferred proton pump inhibitor (PPI) ^16^.

**Figure 1.**
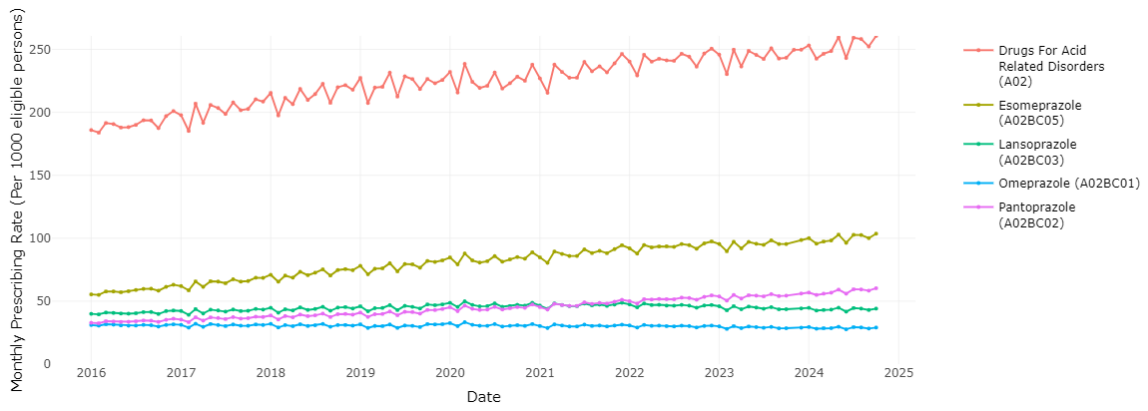
Prescribing rates of individual Proton Pump Inhibitors (PPIs) and drugs for acid related disorders.

This application can be used to visualise the different medications within a therapeutic class, and evaluate trends in use of the HSE preferred drugs over time relative to other agents within the class. Users have the ability to choose various medications from a therapeutic group in order to observe the prescribing trend for each medication and the overall therapeutic group. Examining PPIs as one of the most commonly prescribed therapeutic classes, Figure 1 shows that esomeprazole is the most frequently prescribed PPI, ranging from 55.38 to 103.61 prescription dispensings per 1,000 eligible persons per month, with an increasing trend over time. Pantoprazole, the HSE preferred drug, was the third most frequently dispensed PPI at the start of the period of data coverage (a prescription rate of 32.82 per 1,000 in January 2016), however the rate of prescribing has increased and surpassed lansoprazole since 2021 to reach 60. 26 per 1,000 in October 2024. Esomeprazole is increasing as a proportion of prescribing within drugs for acid-related disorders (from 0.298 in January 2016 to 0.397 in October 2024, Figure 2), while pantoprazole ( Figure 3) increased from 0.177 to 0.231. This indicates improved alignment with the preferred drug initiative recommendation for PPIs, but significant potential for further improvement.

**Figure 2.**
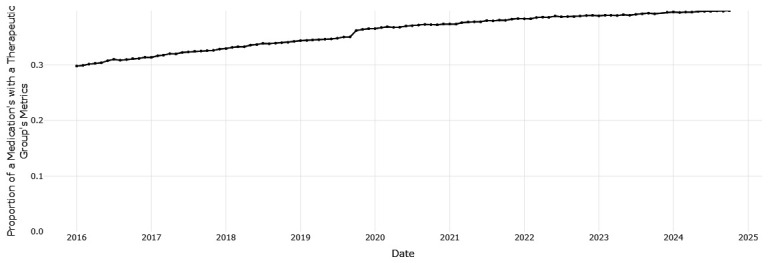
The proportion of prescribing of esomeprazole with all drugs for acid related disorders.

**Figure 3.**
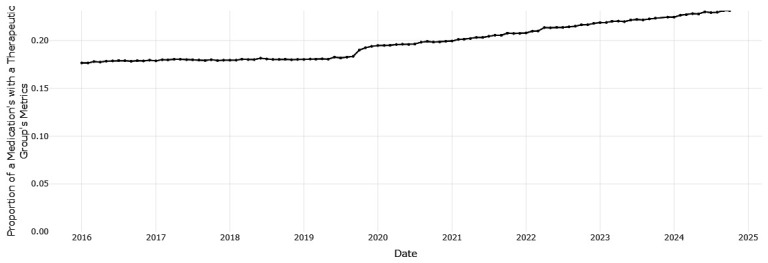
The proportion of prescribing of pantoprazole with all drugs for acid related disorders.

### Cost implications of patent expiry and policy change

The expiry of patent protection and the availability of generic versions of off-patent medications can substantially affect medication expenditure. In Ireland, the Health (Pricing and Supply of Medical Goods) Act 2013 allows pharmacists to substitute medications which have been deemed as interchangeable by the Health Products Regulatory Authority (HPRA) ^17^. ‘Interchangeable products’ are those medications that are judged to be suitable for substitution with another medication in the same group if they have the same qualitative and quantitative composition in each of their active substances, are in the same pharmaceutical form, have the same route of administration, and have no more than two active substances ^17^. Subsequent to the introduction of interchangeable products, the HSE introduced a policy of reference pricing, which involves establishing a common reimbursement price, or reference price, for a group of interchangeable medications ^17^. This is the price the HSE will reimburse each time a product from this group is dispensed; when products are priced at or below this reference price, patients do not face any additional expenses, however dispensing a more expensive product may result in patients having to pay the difference in price ^17^.

**Figure 4.**
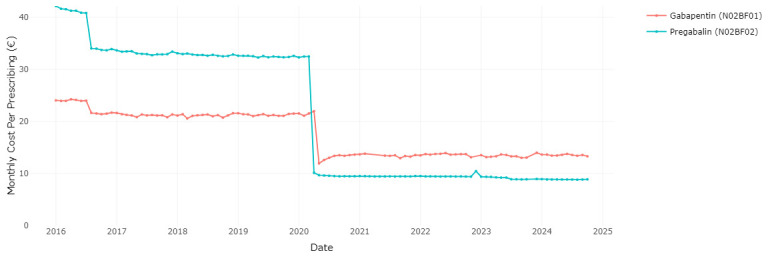
Cost per prescribed unit of gabapentin and pregabalin.

In December 2019, the HPRA published an updated list that included interchangeable pregabalin and gabapentin medications for the first time ^18^. The HSE than set reference prices effective from 1 ^st^ April 2020 for the groups of interchangeable pregabalin products ^19^, and from 1 ^st^ May 2020 for gabapentin products ^20^, in both cases representing a substantial price reduction compared to the previous prices. Figure 4 illustrates the impact of allowing interchangeability between brands and then reference pricing for gabapentinoids. Following these changes, the ingredient cost per dispensing of prescribed pregabalin and gabapentin experienced a considerable decrease. Specifically, the cost for pregabalin dropped from €32.49 per prescription in March 2020 to €10.14 per prescription in April 2020, and the cost for gabapentin dropped from €21.98 per prescription in April 2020 to €11.95 per prescription in May 2020 ( Figure 4).

**Figure 5.**
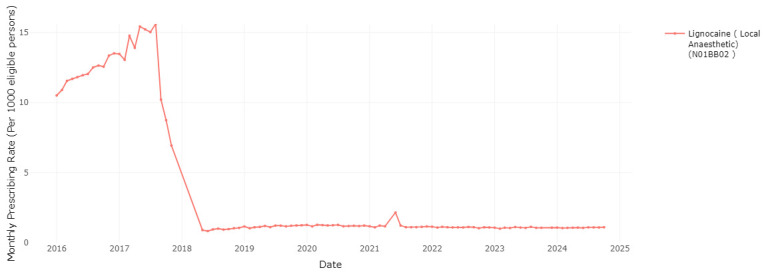
Prescribing rate of lidocaine products (including lidocaine plasters).

As another measure to manage pharmaceutical costs, the HSE introduced a change to the reimbursement of lidocaine plasters, due to a level of use that far exceeded approved indications, indicating off-label prescribing for conditions other than post-herpetic neuralgia (PHN) ^21^. This was implemented in two stages, the initial stage was implemented in September 2017 for new patients, necessitating approval for each patient diagnosed with PHN to be reimbursed under the GMS scheme for lidocaine plaster dispensing. This requirement was subsequently extended to pre-existing patients who had been receiving the drug prior to September 2017 ^21^. As shown in Figure 5, the monthly rate of lidocaine plaster dispensings per 1,000 eligible persons reduced from a high of 15.6 in August 2017 to 10.2 and 8.7 in September and October 2017 following the introduction of the first stage of the policy change. Data are not reported for lidocaine plasters for the period November 2017–April 2018, as it was not among the top 100 medications by frequency or cost, though from May 2018 it is again included in the top 100 by cost. Previous research has shown prescribing frequency decreased further in December 2017 with the introduction of the second stage of the policy change and has remained low since ^22^.

### Seasonal variation in medication prescribing

**Figure 6.**
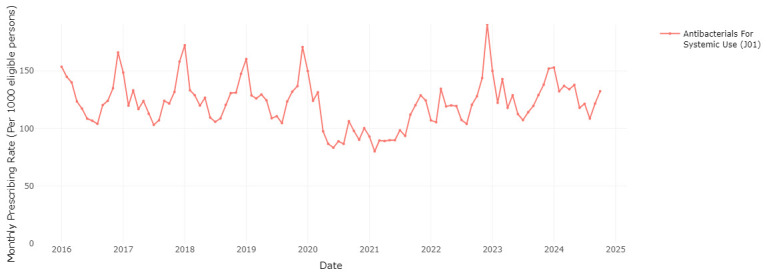
Prescribing rate of the systemic antibacterials therapeutic group.

**Figure 7.**
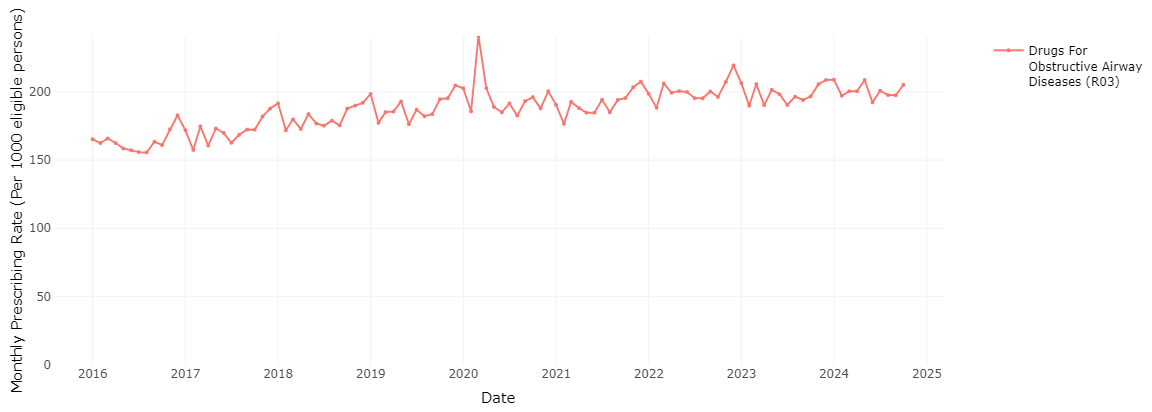
Prescribing rate of the drugs for obstructive airway diseases therapeutic group.

The RxTrends application provides a graphical analysis of prescription trends, presenting monthly data over the course of years, which allows potential seasonality in medication use to be evaluated. Figure 6 and Figure 7 clearly show a considerable increase in prescription rates of antibacterials for systemic use and drugs for chronic obstructive lung disease over the winter months, reaching a peak between December and January of each year. This spike is likely associated with the greater occurrence of infections and respiratory diseases during the colder months (as a possible trigger), requiring higher usage of these medications. The sharp seasonal peaks are less pronounced or absent for the winters of 2020/2021 and 2021/2022, likely due to social distancing and other measures to reduce transmission of COVID-19 during the initial phases of the pandemic ^23^. These measures also reduced circulation of other respiratory infections ^23^.

Similar to data from OpenPrescribing in England, the current application shows a spike in the prescription rate of drugs for chronic obstructive airway diseases (R03) in March-April 2020, coinciding with the onset of the COVID-19 pandemic in Ireland ^24^. This may be attributed to increased patient stockpiling, and improved adherence to inhalers during the initial months of the pandemic due to public health messaging that people with respiratory conditions may be at high risk of complications of COVID-19 ^24^.

## Discussion

The development of the RxTrends application that visualises the patterns of prescribing of medications on the GMS scheme in Ireland provides a useful tool for health data analytics for a range of stakeholders. This application makes use of existing open data that is available on a tabular basis by month, and provides a simple means of interactive visualisation of trends in drug utilisation. The application allows for analysis at multiple levels (individual medication, therapeutic group, or physiological system) and includes a variety of metrics on prescribing frequency, cost, and rates. This provides an advantage over the status quo, where collating and analysing data on a single medication requires downloading and managing a substantial number of monthly files to extract the necessary data, and knowledge and skills to do so. Thus, the RxTrends application increases access to and usability of PCRS data for various audiences it may be of interest to, including researchers, healthcare professionals, policymakers and the general public. As illustrated in the use cases, it has various potential uses, including general assessment of drug utilisation patterns, exploring how utilisation and expenditure vary following policy or other changes, and may provide a means of preliminary evaluation of such policies and scoping potential research questions to generate hypotheses.

OpenPrescribing is another example of interactive data visualisation tool in England that uses data from the English Prescribing dataset (EPD) and the Prescription Cost Analysis (PCA) ^25^. Similar to RxTrends, it allows users to analyse monthly data and time trends in prescribing and costs for individual medications or a group of drugs, as well as the relative metrics of individual medications within therapeutic groups ^25^. Unlike RxTrends, OpenPrescribing includes region-, area- and GP practice-level prescribing and cost data, allowing for analysis across different practices, primary care networks, integrated care boards, or NHS England regions ^25^. Data are reported at the product level, allowing analysis of prescribing by product strength, brand, and formulation. OpenPrescribing also offers additional dashboards for visual insights into over 70 different prescribing measures, such as items which should not be routinely prescribed in primary care, high dose PPIs, and high dose opioids ^25^. Unlike RxTrends that only processes data from 2016 onwards, OpenPrescribing processes an extensive dataset of over 700 million records to visualise long-term trends of annual data for all medications dispensed in community settings in England, extending back to 1998 ^25^.

Researchers have utilised OpenPrescribing data for a variety of studies, for example, to investigate the potential cost reductions by optimising the price-per-unit of drugs and doses within general practices across NHS England ^26^. According to their analysis, it was estimated that a theoretical maximum of £410 million could be saved within a period of 12 months, with half of these potential savings considered practically achievable ^26^. Subsequently, they directly investigated the impact of utilising OpenPrescribing on alterations in prescribing behaviour ^27^ and showed that the platform's price-per-unit tool led to substantial cost savings ^27^. The "price-per-unit" function of OpenPrescribing saved £243,000 at the practice level and £1.47 million at the CCG level over the first three months (August to November 2017) for practices that viewed the feature ^27^. Extrapolating these savings to all practices could yield a national annual NHS saving of £26.8 million ^27^. Outputs from both OpenPrescribing and RxTrends could be combined to facilitate cross-country comparative research, however only for the medications or therapeutic groups included in the latter.

The RxTrends application’s functionality is limited by deficiencies in the source data available. The utilised data encompasses only the top 100 medications by frequency and cost and therapeutic groups prescribed each month. This limits the potential to evaluate prescribing trends of all medications, and prevents evaluation of more granular, specific chemical subgroups (e.g. proton pump inhibitors, ATC A02BC) compared to the available therapeutic groups (e.g. drugs for acid-related disorders, ATC A02). This can also lead to an incomplete time series for specific medications in cases where they drop out of the top 100 medications in particular months during the study period. Furthermore, more detailed information on prescribing of particular products e.g. brands, strengths, or formulations, or prescribing by region or GP practice is not included in the data, as is the case in other jurisdictions. This would allow for more extensive analysis, as is illustrated by OpenPrescribing, and increase the relevance and utility of the application for individual healthcare professionals. While the open data published by the PCRS has facilitated the RxTrends application to be developed, the availability of further data on all prescribed medications, and potentially by product and region/GP practice, would further increase the potential and value of this application and the data in general for informing healthcare research, policy, and practice in Ireland.

While the application's final interface reflected iterative improvements based on feedback from research team members who represent the target audiences, this internal process does not constitute formal user testing. Formal usability testing with external and representative sample of users, including healthcare professionals and other relevant audiencias, would be of benefit to evaluate the user-friendliness of the application and inform improvements in the future.

Notwithstanding these limitations, RxTrends represents the first tool to facilitate visualisation and analysis of open prescribing data in Ireland. While we provide some illustrative use cases, making more extensive data available to be used in the application would enable extensive use for quality improvement initiatives. In particular, availability of practice level data would provide substantial opportunities for clinical audit and feedback, which could result in benefits in prescribing safety, appropriateness, and cost effectiveness. Expanding to include such data has the potential to result in a more effective tool that can play a role in healthcare policy, practice, and research in Ireland, advancing the strategic utilisation of data in supporting high-quality healthcare and patient safety.

## Ethics and consent

Ethical approval and consent were not required.

## Data Availability

Original data obtained for use in this application is available from the PCRS Reporting and Open Data area: https://www.sspcrs.ie/portal/annual-reporting/. Processed data used in this application is available as follows: Zenodo: Data associated with "RxTrends: An R-based Shiny Application for Visualising Open Data on Prescribed Medications in Ireland", https://doi.org/10.5281/zenodo.14726923 ^28^. Data are available under the terms of the Creative Commons Attribution 4.0 International license (CC-BY 4.0).

## Software availability

- Source codes available from: https://github.com/moriarty-pharmacoepi/RxTrends-RShiny
- Archived software available from: https://doi.org/10.5281/zenodo.14726890 ^29^
- License: Available under the terms of the Creative Commons Zero "No rights reserved" data waiver (CC0 1.0 Public domain dedication).
