# Supplementary material for: RxTrends: An R-based Shiny Application for Visualising Open Data on Prescribed Medications in Ireland
Source: HRB Open Res. 2025 May 27;8:36. Originally published 2025 Feb 26. [Version 2] doi: 10.12688/hrbopenres.14080.2 (PMC12130776; doi:10.12688/hrbopenres.14080.2)
Supplement: Peer review history

# Reviewer report: Reviewer response for version 2

**DOI:** 10.21956/hrbopenres.15556.r47489

**Published:** 2025-09-03

**Authors:** Staffan Svensson

**Affiliations:** 1 University of Gothenburg, Gothenburg, Sweden

**Summary**

 This paper describes the background to and development of an R package and application called RxTrends. The intention of the authors is for this to facilitate interpretation of prescription data released monthly by the Irish Health Service Executive. Some examples of its use are given.

 In general the application works well and is visually pleasant. There are some minor quirks that I outline below. The limitations of RxTrends are mainly secondary to the limitations of its source data, as the "top 100" publishing policy of the Irish authority curbs many attempts to follow prescribing trends. The source data also precludes regional comparisons. By making this evident, RxTrends constitutes an impetus for the Irish authority to start releasing full monthly data.

 In the following I make some comments on the sections of the manuscript.

**Introduction**

 For an outsider, it is a bit difficult to digest the information on the PCRS, GMS, DPS, LTI, CCGs, etc. The background on how the Irish system works is necessary, particularly the information about population coverage, but could it be explained in a simpler way? Also, I was looking for a figure along the lines of "These statistics cover X% of all prescriptions filled by the entire Irish population". Perhaps this is deducible from 30.4% × 87% but it would in that case help to have it spelled out.

 The content that is more specific for this article starts with the section "Several countries...". Could the sections be shuffled so this appears earlier?

 The "ingredient cost" as I understand it refers to the price of the actual drug, the rest being costs added by pharmacies. This could be explained.

**Method**

 The description of the application's development is easy to follow. The authors have added data that was not directly available online: eligibility figures for 2016, and ATC codes for individual medications, thus extending the dataset and not merely re-presenting it. It could be made clear that the starting month of January 2016 is due to the PCRS making data available from this time. The most recent data in RxTrends are from December 2024 (writing this in August 2025), while on the PCRS website, data from April 2025 are available. A comment on the updating policy (automatic or manual?) would be in place.

**Use cases**

 The use cases are interesting and well written. Having no experience of looking at Irish prescription data, however, I decided to make my own use cases and compare the function of the PCRS website and the RxTrends application with a source that I am familiar with, the Swedish National Prescribed Drug Register: https://sdb.socialstyrelsen.se/if_lak/val.aspx in order to investigate two topics: 1) use of anastrozole among men, and 2) regional differences in prescription of GLP1-analogues. Topic 1) is due a recent case where a male patient in my clinic explained that he got anastrozole for impotence, from an urologist. Such use of anastrozole is off-label as this drug is normally used to treat breast cancer, and I was interested in knowing how often this is prescribed to men. As for topic 2, the use of GLP1-analogues has boomed and there are clear regional differences in their use in Sweden. Would this be the case also in Ireland?

 Using the Swedish Register, I entered "Monthly numbers" and anastrozole's ATC code "L02BG03", "Nationwide", "Men" and "Number of patients", "All years" and "All months". This gives me a table and graph of the number of men filling anastrozole prescriptions medication since 2006. It seems the number was <20 per month until 2017, then increased to a maximum of 86 in October 2020, then returning to 20--40 monthly prescriptions from 2023 onwards.

 As for the regional comparison of GLP1-analogues, I entered the ATC codes A10BJ01 to 06 as well as A10BX16 for tirzepatide, and with the appropriate settings got a series of graphs showing an explosive development, most notably since 2019 for semaglutide. For this drug, the range of filled prescriptions per 1000 inhabitants in 2024 ranges from 52 in the Västerbotten region, to 149 in the Jämtland region.

 Turning to the PRCR's website https://www.sspcrs.ie/portal/annual-reporting/report/pharmacy I find a page listing the 100 most commonly prescribed products by frequency and cost (anastrozole predictably not being among them) and there is a menu allowing me to add months starting from 2016-01 through 2025-04, but there is no way to see development over time and no regional information.

 Now, starting the RxTrends application in R, after some initial complaints about missing packages, I get a local instance of the Shiny app in my browser. As for the anastrozole question, the nearest I get is data from its second-level ATC group L02 (Endocrine therapy), which is of little use. Regarding the GLP1-analogues, it is possible to see the development over time for semaglutide, liraglutide and dulaglutide on a nation-wide basis, thus clearly improving on the PRCR experience (regional data is obviously still missing). It is shown that semaglutide peaked in late 2022, then decreased, only to rebound in 2024. 

 The program when run from R is fast and responsive when I add more comparators. It's easy to switch between the five tabs and the overall impression is neat. A stress test of the web version (selecting all available medications) made it crash, however. The downloading of data works well.

 When I reproduce the use case of pregabalin and gabapentin, data for both appear to end abruptly in late 2022, but this turns out to be an ATC relabeling issue as these drugs were moved from N03AX (Other antiepileptics) to N02BF (Gabapentinoids) around that time. 

 Playing around with the program, gaps in the time curves are often seen, an artefact due to the "top 100" publishing policy of the PCRS. For example, looking at sodium valproate, data ends in June 2021 although https://www.hpra.ie reports its continued use. The patchy availability of data is somewhat frustrating. When looking at seasonality in use of antihistamines, cetirizine and fexofenadine show a clear pattern of increase in summer. On the other hand, desloratadine and levocetirizine only make it into the top 100 some summers, and the resulting curve with scattered points and lines extrapolated in between give an impression of higher-than-actual use.

 The "compare" function has a potential flaw. It requires the user to choose a drug, say the PPI A02BC05 (esomeprazole) and its corresponding second-level ATC code (A02), and then hitting the "compare" button to get the proportion of A02BC05 within A02. But there is nothing to prevent the user from picking an irrelevant comparator, say L04 instead of A02, thereby generating nonsensical data. It would be an improvement if the application could generate an error message whenever the ATCs do not match, or pick the comparator automatically.

**Discussion**

 The discussion is well written, although the parts that concern OpenPrescribing are unnecessarily long and detailed (mostly the third paragraph starting "Researchers have utilised..."). The point here is to compare RxTrends and OpenPrescribing, and to give some evidence to the usefulness of RxTrends. That could be done in fewer words, perhaps instead using that space to discuss visualisation tools available in other countries.

**Application details**

 At the "Cost" tabs there is reference in the tool tip to a "toggle button above", but this button is actually situated to the left. 

 In the comparison of a medication to its therapeutic group (also commented above), the y axis is reported as "Proportion of a Medication's with a Therapeutic Group's Metrics". Here a word, presumably "prescription", is missing. Anyhow, I suggest this label is changed to "Proportion of a medication's prescribing within its therapeutic group". 

**Manuscript style**

 This sentence and its use of "extended" reminds me of sales language: "The RxTrends application’s capabilities were extended by allowing users to select an unlimited number of entities across multiple categories, including medications, therapeutic groups, and physiological systems.". Why not write something more neutral like "users can select any combination of medications, therapeutic groups and physiological systems."

 Lidocaine, mentioned in a use case, is listed as "lignocaine" in the database. Both are correct but this could be confusing.

**Typos**

 In May 2021, A cyberattack => a cyberattack

 relevant audiencias => audiences

Are the conclusions about the tool and its performance adequately supported by the findings presented in the article?

Yes

Is the rationale for developing the new software tool clearly explained?

Yes

Is the description of the software tool technically sound?

Yes

Are sufficient details of the code, methods and analysis (if applicable) provided to allow replication of the software development and its use by others?

Yes

Is sufficient information provided to allow interpretation of the expected output datasets and any results generated using the tool?

Partly

Reviewer Expertise:

Clinical pharmacology, General practice/family medicine

I confirm that I have read this submission and believe that I have an appropriate level of expertise to confirm that it is of an acceptable scientific standard.

# Reviewer report: Reviewer response for version 2

**DOI:** 10.21956/hrbopenres.15556.r47433

**Published:** 2025-06-02

**Authors:** Heike Vornhagen

**Affiliations:** 1 University of Galway, Galway, Ireland

I appreciate the changes made in relation to references relating to user-friendliness and have no further comments.

Are the conclusions about the tool and its performance adequately supported by the findings presented in the article?

No

Is the rationale for developing the new software tool clearly explained?

Yes

Is the description of the software tool technically sound?

Yes

Are sufficient details of the code, methods and analysis (if applicable) provided to allow replication of the software development and its use by others?

Yes

Is sufficient information provided to allow interpretation of the expected output datasets and any results generated using the tool?

Partly

Reviewer Expertise:

User focused design, data dashboards, data visualisation, user interface design

I confirm that I have read this submission and believe that I have an appropriate level of expertise to confirm that it is of an acceptable scientific standard.

# Reviewer report: Reviewer response for version 1

**DOI:** 10.21956/hrbopenres.15467.r46473

**Published:** 2025-04-18

**Authors:** Heike Vornhagen

**Affiliations:** 1 University of Galway, Galway, Ireland

This paper describes the development of **RxTrends**, an interactive RShiny application designed to visualise and explore trends in prescription data under Ireland’s General Medical Services (GMS) scheme. Drawing from publicly available monthly datasets published by the Health Service Executive's Primary Care Reimbursement Service (HSE-PCRS), the tool enables dynamic analysis of prescribing patterns, including frequency, cost, and population-adjusted rates, from 2016 to 2024. The data were cleaned and structured using Python in JupyterLab and integrated into the app using R and various packages like ggplot2, plotly, and shinyjs. The application allows users to compare trends across medications, therapeutic groups, and physiological systems using the WHO’s Anatomical Therapeutic Chemical (ATC) classification.

 RxTrends aims to address a major limitation of the Irish prescribing data—lack of user-friendly access to visualisations—and provides functionality similar to tools like OpenPrescribing in the UK. Use cases illustrated include monitoring adherence to the HSE Preferred Drugs Initiative and evaluating the financial impact of policy changes such as reference pricing and generic substitution.

**Is sufficient information provided to allow interpretation of the expected output datasets and any results generated using the tool?**

 Sufficient information is provided to understand the general nature of the outputs generated by the tool, such as candidate match scores and ranked lists. However, the paper lacks detail on the exact structure, format, or schema of these output datasets, which may limit a reader’s ability to fully interpret or reuse the results. Including output specifications would improve transparency and reproducibility.

**Are the conclusions about the tool and its performance adequately supported by the findings presented in the article?**

 While the paper provides a technical description of the tool and demonstrates its capabilities through examples, the conclusions about its usability and user-friendliness are not sufficiently supported by evidence. There is no mention of user testing or evaluation with end-users, nor is there any indication of user research conducted prior to development to establish need or inform design choices. Without this, it is difficult to assess whether the tool effectively meets user requirements or delivers on its claims of intuitiveness and practicality.

**Recommendation: Approve with Reservations**

 The tool presents a promising approach to visualising healthcare data, with a well-executed implementation and clear presentation of results. However, I have significant reservations regarding the tool's user-centric design and its lack of clear user research. While the authors mention the tool is ‘user-friendly,’ there is little to no evidence presented of user testing or engagement that would support such claims. Additionally, there is no indication of a structured process to assess user needs prior to development, which raises concerns about how well the tool truly aligns with its intended audience’s requirements.

 Further, the conclusions about the tool’s performance and usability seem premature without substantial user feedback. To strengthen this work, I recommend more thorough user testing and a detailed explanation of the methodology behind understanding user needs, which would support the tool’s claims of being intuitive and effective.

 If these areas are addressed, this tool has the potential to be a valuable resource.

Are the conclusions about the tool and its performance adequately supported by the findings presented in the article?

No

Is the rationale for developing the new software tool clearly explained?

Yes

Is the description of the software tool technically sound?

Yes

Are sufficient details of the code, methods and analysis (if applicable) provided to allow replication of the software development and its use by others?

Yes

Is sufficient information provided to allow interpretation of the expected output datasets and any results generated using the tool?

Partly

Reviewer Expertise:

User focused design, data dashboards, data visualisation, user interface design

I confirm that I have read this submission and believe that I have an appropriate level of expertise to confirm that it is of an acceptable scientific standard, however I have significant reservations, as outlined above.

# Response

**Published:** 2025-05-23

**Authors:** Ahmed Hassan Ali

**Affiliations:** Royal College of Surgeons in Ireland 26 York Street Campus, Dublin, Leinster, Ireland

Thank you for your thoughtful and constructive feedback. We have carefully considered each of your comments and revised the manuscript accordingly, as detailed below.

**Reviewer Point 2.1** – lacks detail on the exact structure, format, or schema of these output datasets, which may limit a reader’s ability to fully interpret or reuse the results.

**Reply:** We thank the reviewer for identifying this gap. We have expanded the description of dataset outputs both in a new Help and documentation section of the application, and within the manuscript itself: “Downloadable data is provided in Comma-separated values (CSV) format, including columns for the metrics listed in Table 1 and one row per month per selected medication, therapeutic group, and/or physiological system. A contextual help and documentation feature has also been embedded within the application, which describes output data format in detail.”

**Reviewer Point 2.2** – Lack of evidence for usability and user-friendliness claims and lack of indication of user research conducted prior to development to establish need or inform design choices, or evaluation with end-users

**Reply:** We acknowledge this is a valid concern. We have revised the manuscript to remove references to user-friendliness as this has not been formally assessed.

 While formal user testing was not conducted, the development process did involve testing and feedback on prototypes by the inter-disciplinary research team, which we now describe in the manuscript as follows: “During the development of RxTrends, the application underwent two refinement cycles, with team members (from clinical, epidemiology, and data science backgrounds) evaluating a prototype application and providing feedback on features, functionality, and presentation.”

 Lastly, we have noted lack of formal usability testing as a limitation and something beneficial for further development of the application: “While the application's final interface reflected iterative improvements based on feedback from research team members who represent the target audiences, this internal process does not constitute formal user testing. Formal usability testing with external and representative sample of users, including healthcare professionals and other relevant audiences, would be of benefit to evaluate the user-friendliness of the application and inform improvements in the future.”

# Reviewer report: Reviewer response for version 1

**DOI:** 10.21956/hrbopenres.15467.r46472

**Published:** 2025-04-10

**Authors:** Marion Bennie, Barry Melia, Amanj Kurdi

**Affiliations:** 1 University of Strathclyde, Glasgow, UK; 2 Public Health Scotland, Scotland, UK

This article outlines how standard R packages, by importing a limited number of data rows, can be utilised to provide medicines utilisation intelligence. As highlighted in the article, this is limited to the top 100 lines in the GMS scheme alone. I agree that this limits the functionality significantly (whilst DPS information may not be comprehensive, it would be good to present and caveat as these patients might be considered as high-cost families). I agree that future functionality, broken down geographically/organisationally would enhance the offering. I would also suggest that some quality prescribing metrics beyond the preferred drugs list would add a medicines safety aspect which would allow benchmarking and quality improvement. 

 However, as a first exploration, this is good start. I would recommend a discussion with colleagues in England and Scotland to further explore the “art of the possible”. Thank you and well done!

Are the conclusions about the tool and its performance adequately supported by the findings presented in the article?

Yes

Is the rationale for developing the new software tool clearly explained?

Yes

Is the description of the software tool technically sound?

Yes

Are sufficient details of the code, methods and analysis (if applicable) provided to allow replication of the software development and its use by others?

Yes

Is sufficient information provided to allow interpretation of the expected output datasets and any results generated using the tool?

Yes

Reviewer Expertise:

NA

We confirm that we have read this submission and believe that we have an appropriate level of expertise to confirm that it is of an acceptable scientific standard.

# Response

**Published:** 2025-05-23

**Authors:** Ahmed Hassan Ali

**Affiliations:** Royal College of Surgeons in Ireland 26 York Street Campus, Dublin, Leinster, Ireland

We thank reviewers for their thoughtful feedback on our manuscript. Below, we address each of their comments and outline the amendments made to strengthen our paper.

**Reviewer Point 1.1** – Limitation to top 100 lines in GMS scheme and suggestion to include DPS information

**Reply:** We appreciate the reviewer highlighting this limitation. In response, we have expanded our data coverage to include DPS (Drugs Payment Scheme) and other scheme information with appropriate caveats noting that does not capture comprehensive data. These additional data are now integrated into an expanded version of the application. However, this expanded version does not show interactive graphs for prescribing/cost rates, as the denominators (i.e. eligible numbers) are inconsistent for schemes other than the GMS scheme.

**Reviewer Point 1.2** – Geographic/organizational breakdown would enhance the offering and quality prescribing metrics beyond the preferred drugs list

**Reply:** We agree that geographic and organisational breakdowns would significantly enhance the utility of the tool. We have noted this as a priority for inclusion in future iterations of RxTrends, once relevant data is available. The reviewers also make an excellent suggestion regarding quality prescribing metrics. Based on the currently available data, only metrics relating to the volume of prescribing can be generated; however, availability of more detailed data on individual products and strengths prescribed may allow expansion to other quality metrics in the future.

**Reviewer Point 1.3 –** Discussion with colleagues in England and Scotland

**Reply:** Thank you for the suggestion. We have already discussed this with colleagues in England and we hope to discuss further with other colleagues once more data becomes available allowing expansion of the functionality.
